# Targeted Ablation of Primary Cilia in Differentiated Dopaminergic Neurons Reduces Striatal Dopamine and Responsiveness to Metabolic Stress

**DOI:** 10.3390/antiox10081284

**Published:** 2021-08-13

**Authors:** Rasem Mustafa, Chahinaz Rawas, Nadja Mannal, Grzegorz Kreiner, Björn Spittau, Katarzyna Kamińska, Rüstem Yilmaz, Christina Pötschke, Joachim Kirsch, Birgit Liss, Kerry L. Tucker, Rosanna Parlato

**Affiliations:** 1 Institute of Anatomy and Cell Biology, Heidelberg Medical Faculty, University of Heidelberg, 69120 Heidelberg, Germany; rasem.mustafa@najah.edu (R.M.); joachim.kirsch@uni-heidelberg.de (J.K.); ktucker2@une.edu (K.L.T.); 2 Institute of Applied Physiology, Ulm Medical Faculty, University of Ulm, 89081 Ulm, Germany; chahinaz-rawas@uni-ulm.de (C.R.); nadja.mannal@uni-ulm.de (N.M.); christina.poetschke@uni-ulm.de (C.P.); birgit.liss@uni-ulm.de (B.L.); 3 Department of Brain Biochemistry, Maj Institute of Pharmacology, Polish Academy of Sciences, Smetna 12, 31-343 Kraków, Poland; kreiner@if-pan.krakow.pl; 4 Institute of Anatomy and Cell Biology, Department of Molecular Embryology, Faculty of Medicine, University of Freiburg, 79104 Freiburg, Germany; bjoern.spittau@uni-bielefeld.de; 5 Anatomy and Cell Biology, Medical School OWL, Bielefeld University, 33615 Bielefeld, Germany; 6 Department of Pharmacology, Maj Institute of Pharmacology, Polish Academy of Sciences, Smetna 12, 31-343 Kraków, Poland; katarzyna1.kaminska@uj.edu.pl; 7 Jagiellonian Center for Experimental Therapeutics, Jagiellonian University, Bobrzynskiego 14, 30-348 Kraków, Poland; 8 Mannheim Center for Translational Neuroscience, Division of Neurodegenerative Disorders, Department of Neurology, Mannheim Medical Faculty, University of Heidelberg, 68167 Mannheim, Germany; ruestem.yilmaz@medma.uni-heidelberg.de; 9 Linacre College and New College, University of Oxford, Oxford OX1 2JD, UK; 10 Department of Biomedical Sciences, College of Osteopathic Medicine, Biddeford, ME 04005, USA; 11 Center for Excellence in the Neurosciences, University of New England, Biddeford, ME 04005, USA

**Keywords:** primary cilium, intraflagellary protein, substantia nigra, striatum, dopamine, pacemaker, multielecotrode arrays (MEA), D2-autoreceptor

## Abstract

Primary cilia (PC) are microtubule-based protrusions of the cell membrane transducing molecular signals during brain development. Here, we report that PC are required for maintenance of Substantia nigra (SN) dopaminergic (DA) neurons highly vulnerable in Parkinson’s disease (PD). Targeted blockage of ciliogenesis in differentiated DA neurons impaired striato-nigral integrity in adult mice. The relative number of SN DA neurons displaying a typical auto-inhibition of spontaneous activity in response to dopamine was elevated under control metabolic conditions, but not under metabolic stress. Strikingly, in the absence of PC, the remaining SN DA neurons were less vulnerable to the PD neurotoxin 1-methyl-4-phenyl-1,2,3,6-tetrahydropyridin (MPTP). Our data indicate conserved PC-dependent neuroadaptive responses to DA lesions in the striatum. Moreover, PC control the integrity and dopamine response of a subtype of SN DA neurons. These results reinforce the critical role of PC as sensors of metabolic stress in PD and other disorders of the dopamine system.

## 1. Introduction

Primary cilia (PC) are non-motile microtubule-based cellular antennas that arise at the plasma membrane of most mammalian cell types during growth arrest [1,2]. PC possess an axoneme, a microtubular structure that extends from the basal body and it is surrounded by a specialized plasma membrane. They also include axoneme-based transport machinery called intraflagellar transport (IFT), which uses different motor protein complexes for anterograde versus retrograde transport [3]. Due to their small volume and high enrichment in membrane receptors, PC represent a crucial hub for the coordination of multiple signaling pathways, and play a critical role in tissue differentiation and homeostasis [2]. PC in differentiated cells have been reported to be important for the response to changes in glucose concentration. In particular, when PC are disrupted, intrinsic activities of pancreatic beta-cells are dysregulated because of abnormal glucose sensing [4]. Similarly, substantia nigra (SN) dopaminergic (DA) neurons also appear to be sensitive to changes in the extracellular glucose concentration, increasing their activity in high glucose [5]. In addition, previous data based on systemic glucose injections in rats also show an inhibitory effect on the firing of SN DA neurons [6]. Type II diabetes characterized by increased blood glucose and insulin resistance seems to increase the risk to develop Parkinson’s disease (PD), an age-related neurodegenerative disease in which SN DA neurons are progressively lost [7,8]. Several studies indicate a ciliary role in the function of the dopamine system. D1-type dopamine receptors (D1Rs) are G-protein-coupled receptors localized to the primary cilium of different cell types, suggesting a ciliary role in dopamine signaling transmission [9,10]. Increased PC length has been reported in various models of Huntington’s disease (HD) and HD patients, characterized by progressive degeneration of GABAergic striatal medium spiny neurons (MSNs) that receive glutamatergic and DA synaptic inputs [11,12,13,14,15,16]. The DA input originates from DA midbrain neurons. In various pharmacological and genetic models of PD, the lack of DA inputs results in elongated PC in the striatum [17,18,19,20,21,22]. Notably, the PD pathogenic protein kinase leucine-rich repeat kinase (LRRK2) suppresses PC formation [18,19]. Despite this evidence, the functional link between PC and progressive age-related neurodegenerative disorders affecting the dopamine system remains underexplored. 

This work investigates the consequences of oxidative stress triggered by a mitochondrial complex I inhibitor, the neurotoxin MPTP, on the PC in the dopamine system. The MPTP model is relevant to pharmacologically mimicking PD in mice. Recent work showed a crosstalk between mitochondrial function and ciliogenesis [23]. Changes in the length of PC have been reported in various cellular systems and organs (e.g., kidney, thyroid gland, brain) in response to several oxidative stress triggers, including H_2_O_2_ [22,24,25].

With the Cre-loxP system, we have previously achieved the conditional ablation of the *Ift88* gene, an anterograde component of the IFT complex system, in cells expressing the transcription factor engrailed homeobox 1 [26,27]. This mutation resulted in the loss of PC in the developing midbrain prior to expression of markers of DA differentiation, leading to a reduced number of differentiated ventral midbrain DA neurons at late embryonic stages [26,27]. As these mutant mice were perinatally lethal, the role of PC in postnatal survival and during aging could not be investigated. More recently, by the ablation of *Ift88* in dopaminoceptive neurons, we have shown that PC are dispensable for maintenance of D1R expression in striatal neurons; however, striatal content of dopamine metabolites increases in aged mutant mice, supporting a PC-dependent functional crosstalk between dopaminoceptive and DA neurons [16]. 

To specifically address the cell-autonomous functions of PC for the maintenance of differentiated DA neurons, with the Cre-loxP recombination system we generated a mouse model in which the *Ift88* gene is selectively ablated upon expression of the dopamine transporter at E13.5 [28,29]. Notably, loss of PC reduced DA projections, dopamine content in the striatum, and the number of SN DA neurons. Moreover, the lack of PC resulted in a decreased number of dopamine-excited SN DA neurons under control metabolic conditions. By this genetic approach, mutant mice lacking PC in DA neurons were not responsive to the PD-inducing toxin 1-methyl-4-phenyl-1,2,3,6-tetrahydropyridine (MPTP). However, the length of PC in striatal neurons was increased in response to DA deficit in both the conditional *Ift88* mutant and in the MPTP-treated mice.

## 2. Materials and Methods

### 2.1. Mice 

Procedures involving animal care were approved by the Committee on Animal Care and Use (Regierungspräsidium Karlsruhe, Germany) in accordance with the local Animal Welfare Act and the European Communities Council Directives (2010/63/EU and 2012/707/EU). MPTP-induced neurodegeneration in mice was performed after approval of the Official Animal Welfare Officer of the Center for Experimental Models and Transgenic Services (CEMT) at the University of Freiburg, Germany. All animal procedures were conducted in accordance with local ethical guidelines and have been approved by the Animal Experimentation Committee of the University Freiburg, and the Regierungspräsidium Freiburg, or Tübingen, Germany (G-15/111). Mice were housed with a standard 12 h light/dark cycle and with ad libitum access to food and water.

To generate conditional knock-out mice lacking IFT88 in DA neurons, we deleted exons 4–6 of the *Ift88* gene with the Cre-LoxP system [30]. We crossed mice homozygous for the loxP-flanked (floxed) *Ift88* allele with heterozygous transgenic mice that expressed the Cre recombinase under the control of the dopamine transporter (DAT) genetic locus [28]. Double heterozygous mutant mice were further crossed with homozygous floxed *Ift88* mice to obtain mice homozygous for the floxed *Ift88* allele and heterozygous for the DAT::Cre transgenic allele (25% of the offspring, *Ift88^DAT::Cre^*, abbreviated: *Ift88* cKO) (mouse strain official nomenclature: B6.CBA-*Ift88*tm1.1BkyTg(DAT::Cre)9076Gsc). These mutant mice were born at the expected Mendelian ratio, they showed normal lifespan, and no gross abnormalities (observed until 6 months).

Ear biopsies were used for genotyping of DAT::Cre and floxed *Ift88* alleles by PCR, as previously reported [28,30]. 

### 2.2. Brain Dissection and Tissue Preparation for Histology and Western Blot

For histological analysis, after transcardial perfusion with 4% PFA/PBS (pH 7.2), mouse brains were washed in PBS (pH 7.2) and cryoprotected by incubating them overnight at 4 °C in 10%, 20%, and 30% sucrose, respectively. The brains were frozen by coronal orientation in tissue freezing medium (TissueTek, Leica, Wetzlar, Germany), using a mixture of liquid nitrogen and dry ice, and were stored at −80 °C until sectioning by a cryostat (CM3050S, Leica). Free-floating sections (30 μm) were collected in 48-well plates and stored at 4 °C in PBS until immunostaining, while cryosections (12 μm) on SuperFrost Ultra Plus glass slides (Thermo Scientific, Waltham, MA, USA) were stored at −80 °C. For the analysis of embryonic brains, we used for PC frequency coronal sections from midbrain (30 μm) and, for TH immunoreactivity, coronal sections from striatum (12 μm), both mounted serially on SuperFrost Ultra Plus glass slides. For the analysis of adult brains, we used either coronal sections from striatum (12 μm) mounted on glass slides for TH and DAT immunoreactivity or free-floating striatum and midbrain sections (30 μm) for analysis of PC frequency and length and for stereology. For the embryonic analysis, we analyzed for striatum the regions between the Bregma coordinates 1.20 mm and 2.16 mm and, for midbrain, 3.36 mm and 4.20 mm [31]. For the adult striatum, we analyzed the regions +0.98 mm and +0.02 mm and, for the midbrain, −2.80 mm and −3.88 mm, according to the adult mouse brain atlas [32]. For Western blots, mouse brains were isolated without transcardial perfusion with 4% PFA, and incubated in 30% sucrose/PBS (pH 7.2) overnight at 4 °C before freezing in tissue freezing medium. Coronal cryosections (300 μm) were cut from one hemisphere, and punches of the dorsal striatum from three sections were obtained by using a Harris Uni-Core (0.75 mm) (Ted Pella, Inc.) based on a previously described procedure [21]. 

### 2.3. Immunohistochemistry

For immunofluorescence, slide-mounted cryosections were thawed for 15 min at room temperature. Both free-floating and on-slide cryosections were blocked with 5% normal swine serum (NSS) (S-4000, Vector), and diluted in PBS pH7.2 with 0.2% Triton X100 (PBST) or only in PBS, respectively, for 30 min at room temperature. Similarly, primary antibodies were diluted in 5% NSS and sections were incubated overnight at 4 °C. Nuclei were stained with 4′,6-diamidino-2-phenylindole (DAPI) (1:10^6^ in PBS, 62248, Invitrogen) for 10 min at room temperature. Slides were mounted with a coverslip using AquaPolymount (18606-20, Polysciences, Hirschberg, Germany). For immunohistochemistry followed by stereological analysis, free-floating cryosections (30 μm) were washed in PBS, and endogenous peroxidases were blocked in 1% H_2_O_2_ diluted in PBS/methanol (1:1) for 15 min. Unspecific binding was prevented by incubation in 5% NSS in PBST for 30 min. The sections were incubated overnight at 4 °C with the TH antibody diluted in 5% NSS in PBST (1:2000, AB1542, Merck Millipore, Burlington, MA, USA ) followed by incubation with biotinylated anti-sheep secondary antibody (1:400, Vector Laboratories Inc., Burlingame, CA, USA) and a Vectastain Elite ABC-HRP (horseradish peroxidase) kit (PK-6100, Vector Laboratories), both diluted in PBST using diaminobenzidine as a substrate for detection (Sigma-Aldrich, St. Louis, MI, USA). The free-floating sections were then mounted on glass slides for microscopy, dehydrated, and coverslips were mounted with Eukitt mounting medium.

Primary antibodies for immunostaining were: sheep tyrosine-hydroxylase (TH) antibody (1:500 for immunofluorescence, AB1542, Merck Millipore, Burlington, MA, USA), rat dopamine transporter (DAT) antibody (1:500, MAB369, Merck Millipore, Burlington, MA, USA), rabbit IFT88 antibody (1:500, gift from Dr. G. Pereira (German Cancer Research Center, Heidelberg, Germany), mouse NeuN antibody (1:100, MAB377, Millipore), rabbit adenylcyclase III (ACIII) antibody (1:500, SC588, Santa Cruz Biotechnology, Dallas, TX, USA), mouse centrosomal protein 43/FGFR1 10P (CEP43/FOP) (1:600, H00011116-M01, Abnova, Taipeh, Taiwan), rabbit ADP-ribosylation factor-like 13B (ARL13B) (1:100, 17711-1-AP, Proteintech, St. Leon-Rot, Germany). For confocal microscopy, appropriate secondary antibodies were used: donkey anti-sheep (1:1000, A-11016, Thermo Scientific, Waltham, MA, USA), donkey anti-rabbit (1:1000, A-21206, Thermo Scientific), donkey anti-mouse (1:100, A-21203, Thermo Scientific), chicken anti-rat (1:100, A-11077, Thermo Scientific), donkey anti-sheep (1:500, A21448, Thermo Scientific), marked with the fluorophores Alexa 594, Alexa 488, Alexa 568, or Alexa 647.

### 2.4. Analysis of Primary Cilia Frequency and Length and TH and DAT Immunoreactivity 

For PC frequency and length quantification, we used a Leica SP8 confocal microscope with a 63x oil immersion objective and maximal intensity projection z-stacked images (1 µm interval), as previously described [16,17]. To determine the number of DA neurons showing primary cilia, on average, 240 TH positive neurons per mouse were analyzed in five non-consecutive 30 μm sections (one every four sections). The percentage of TH positive neurons showing axonemal adenylcyclase III (ACIII) antibody immunostaining was calculated per mouse. Cilia lengths were measured using the free-hand line selection tool of ImageJ software [33]. The graphical and statistical analysis was performed using GraphPad Prism 7.0 (GraphPad Software, San Diego, CA, USA). In general, 80 cells per mouse were analyzed after tracing the ACIII signal in the ImageJ software in line with established protocols [16,17,34]. 

For analysis of TH and DAT immunoreactivity in the dorsal striatum, we acquired images with a 20x oil immersion objective for single planes using a Leica SP8 confocal microscope. To determine TH and DAT immunoreactivity, the optical fiber density was measured in cryosections (12 μm) with ImageJ software. The quantification was performed in eight coronal serial sections per mouse on 8-bit images (grayscale). To measure the mean optical intensity the “mean gray value” was determined. The immunoreactivity was measured by subtracting the mean gray value of the respective background in the cortex from the mean gray value of dorsal striatum [29]. The measurements were limited to the drawn region of interest and the same area was selected in all sections (0.05 mm^2^) [16]. The analysis was performed by an experimenter blinded for genotype and age. The mean values per mouse were then plotted and analyzed for statistical significance as indicated in figure legends and in the supplementary statistical information.

### 2.5. HPLC Analysis of Dopamine Content

The tissue levels of noradrenaline (NA), dopamine, 5-hydroxytryptamine (serotonin; 5-HT), 3,4-dihydroxyphenylacetic acid (DOPAC), and homovanillic acid (HVA) were measured using high-performance liquid chromatography with electrochemical detection (HPLC-EC). The concentrations of endogenous dopamine and its metabolites (DOPAC, HVA) were measured using HPLC-EC, according to a previously described method [35]. Briefly, sections including striatum and cortex were isolated by the mouse brain matrix in the region between Bregma 1.42 mm and −0.10 mm. Average tissue weight was approx. 50 mg. After weighing, the tissue samples were deep frozen in dry ice, stored at −80 °C until further use, and processed as previously described [16]. 

### 2.6. Western Blot Analysis

Tissue punches of dorsal striatum were homogenized in Pierce RIPA buffer (89900, Thermo Scientific) for total protein extraction and Western blot analysis using Bolt 4–12% Bis-Tris Plus precasted gels (NW04122BOX, Thermo Scientific). Primary antibodies were: DAT (1:5000), TH (1:5000) (both incubated overnight), mouse GAPDH (1:80,000, 60004-1-Ig, Proteintech) incubated for 2 h. Secondary antibodies were: goat anti-rat horseradish peroxidase (HRP) conjugated (1:2000, A10549, Invitrogen), donkey anti-sheep HRP conjugated (1:10,000, A16047, Invitrogen), goat anti-mouse HRP conjugated (1:10,000, G21040, Thermo Scientific). Band intensity was normalized to GAPDH signal. The average values were expressed in arbitrary units, as percentage of control mean values.

### 2.7. Electrophysiological Multi-Electrode Array (MEA) Recordings

Vital brain slices from adult mice were prepared, MEA recordings were performed, and MEA data were analyzed essentially as previously described [5]. Briefly, after brain removal, coronal midbrain slices (200 μm) were prepared using a vibratome (Leica VT1200S, amplitude 1.4 mm, speed 0.6 mm/s) with ceramic blades (Campden Instruments Ltd.) in ACSF at ~36 °C. ACSF contained (in mM): 124 NaCl, 3 KCl, 26 NaHCO_3_, 1.2 KH_2_PO_4_, 1 MgCl_2_, 1 CaCl_2_, 10 glucose, bubbled with carbogen (95% CO_2_, 5% O_2_) for pH adjustment (7.3) and oxygenation. Slices were allowed to recover for 1 h at 37 °C in ACSF recovery solution containing (in mM): 125 NaCl, 2.5 KCl, 25 NaHCO_3_, 1.25 NH_2_PO_4_, 1 MgCl_2_, 1 CaCl_2_, 25 glucose. The ACSF for MEA recordings (“recording-ACSF”) contained (in mM): 124 NaCl, 3 KCl, 1.2 KH_2_PO_4_, 26 NaHCO_3_, 1 MgCl_2_, 1 CaCl_2_, and 25 glucose, or 1 glucose and 24 sucrose. We added 10 μM DNQX disodium salt (Tocris) and 10 μM gabazine (Tocris) to block fast synaptic transmission. ACSF was bubbled with 95% CO_2_, 5% O_2_ for pH 7.3 adjustment and oxygenation (osmolarity: 300–315 mOsm/L). MEA recordings were carried out using 3D-Biochips, manufactured by Qwane Biosciences SA (Switzerland), in either a USB MEA1060-Up-BC System or an MEA2100-2x60 System (Multichannel Systems, Reutlingen, Germany), at a bath chamber temperature of 31–33 °C. Data were sampled at 20 kHz using the MC Rack software (version 4, Multichannel Systems, Germany), using a low pass (3000 Hz) and a high pass (200 Hz) Butterworth 2nd order filter. The experimental design was as follows: 10 min baseline recording of pacemaker activities (for determining basal frequency and CV-ISI values), followed by bath application of 100 μM dopamine hydrochloride for 15 min, and a 20 min wash-out phase of dopamine. MEA electrodes that recorded a response to dopamine (i.e., change in event rate) were marked during the recording, based on the online analysis options in the MC Rack software. Spike sorting and cluster analysis of MEA recordings were carried out with Spike2 software (8.02e x64, Cambridge Electronic Design Ltd., Cambridge, UK). After Spike2 analysis, all recordings with identified single unit activity (SUA) were imported into Neuroexplorer software (vers. 4.032, Plexon Inc., Dallas, TX, USA) for generation and analysis of autocorrelation and cross-correlation probability of all recorded SUAs for all electrodes. Mean firing rates (in 20 s bins) of each verified SUA were exported into Excel. To define the basal firing rate, the mean firing rate of a stable 10 min control period at the beginning of each recording was calculated. As a measure of pacemaker regularity, the mean interspike interval (ISI = 1/frequency) and its standard deviation (SD) were determined as previously described [5]. For analysis of firing rates in dopamine, firing rates in the last minute in dopamine (i.e., minute 15) were normalized to the mean firing rate during the 10 min control period. An inhibitory dopamine response was classified as desensitized if the mean frequency in the last minute of dopamine was higher than 5% of the respective basal firing rate.

### 2.8. MPTP Treatment

The neurotoxin 1-Methyl-4-phenyl-1,2,3,6-tetrahydropyridin (MPTP) was used according to the safety guidelines [36]. The injection of MPTP has been described previously [37]. Briefly, mice were intraperitoneally injected with 20 mg/kg of MPTP hydrochloride (Sigma-Aldrich, St. Louis, MI, USA) dissolved in 0.2 mL phosphate-buffered saline (PBS) once a day for three consecutive days. PBS alone was injected in the control group. Mice were sacrificed 1 day after the last injection by cervical dislocation and brains were isolated for further analysis.

### 2.9. Stereological Analysis

The total number of ventral midbrain DA neurons in both SN and ventral tegmental area (VTA) regions was analyzed by stereology using a bright-field microscope (Axioskop^®^ 40, Carl Zeiss AG, Oberkochen, Germany) with a camera (Microfire^®^ TM A/R, Optronics, Fremont, CA, USA), and the Stereoinvestigator software (MicroBrightField, Inc., Williston, VT, USA). The analysis was performed using every second midbrain section corresponding to 15 coronal sections per mouse. The “optical fractionator probe” was selected. The section cut thickness was 30 µm. The probe configurations were defined to measure the thickness of the section at each side. The “size of the counting frame” was defined as x = 45 µm, y = 45 µm (approximately 1–3 cells in each site). The “guard zones” (1–5 µm thick) were excluded because they may include distortions. The depth at which the cells were counted (the dissector height) was set into 15 µm. The Gundersen coefficient of error (CE) was between 0.04 and 0.07 (for m = 1 class) for the analyzed mice, which is considered adequate for this analysis [38]. The experimenter was blinded to genotype and treatment.

### 2.10. Statistical Analysis

The data in the graphs are presented as a mean based on the number of mice ± S.E.M, if not stated otherwise. The individual values are included in the bar graphs. The diagram representation and the statistical analysis were performed using GraphPad Prism software. Statistical significance of the observed differences was assessed by unpaired Student’s *t*-tests, and by two-way analysis of variance (ANOVA) followed by Fisher’s LSD test for normally distributed datasets, and by unpaired Mann–Whitney U tests or by a chi-square test for non-normally distributed datasets, as specified in the respective figure legends and in detail in the supplementary statistical information. 

## 3. Results

### 3.1. Generation of Mutant Mice Lacking Primary Cilia in Differentiated DA Neurons

**Figure 1:**
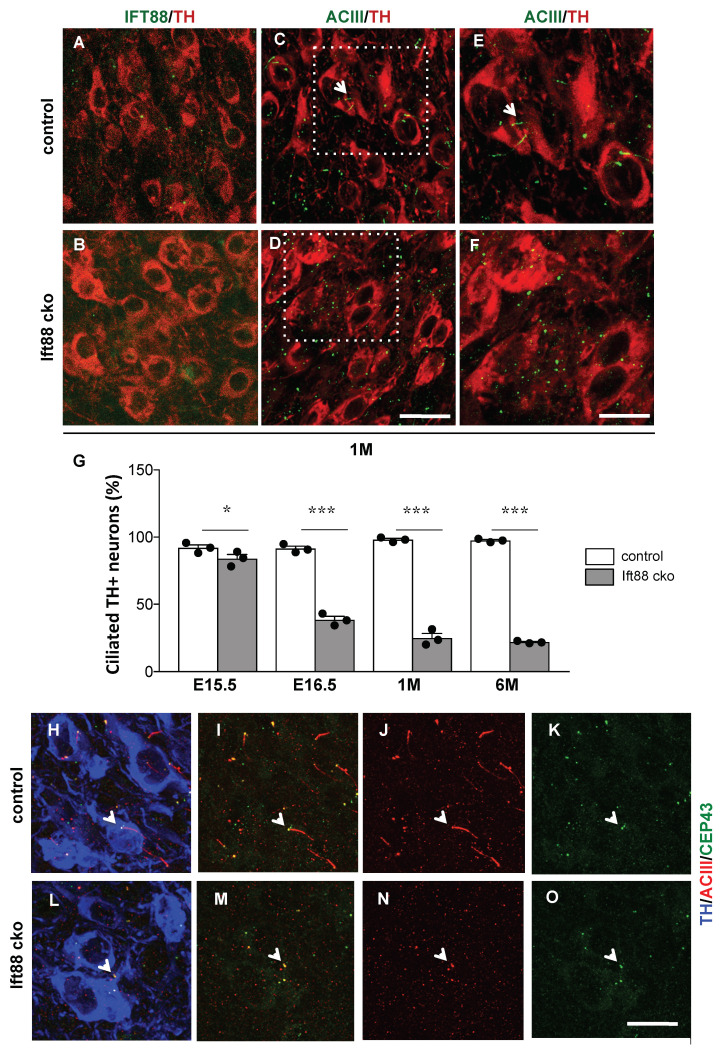
**Conditional ablation of *Ift88* results in the loss of primary cilia in ventral midbrain DA neurons.** (**A**,**B**) Representative confocal images of coronal midbrain sections at 1 month showing loss of IFT88 protein in DA neurons by immunofluorescence with an IFT88-specific antibody (green) and a TH-specific antibody (red), as a marker of DA neurons. (**C**,**D**) Representative confocal images of coronal midbrain sections at 1 month showing loss of primary cilia by immunofluorescence with the marker ACIII (green) in TH immunopositive DA neurons (red) of *Ift88* cKO mice in comparison with control littermates. (**E**,**F**) High magnification of the boxed areas in C and D, respectively, shows loss of the axonemal ACIII signal in the *Ift88* cKO. Arrow points at PC in control mice. (**G**) Quantification of the percentage of TH immunopositive ventral midbrain neurons showing ACIII at different embryonic stages and in young and adult mice (*n* = 3 mice per group). The data are presented as the mean ± SEM. Statistical significance between the indicated groups determined by two-way ANOVA followed by Fisher’s LSD test. * *p* < 0.05, *** *p* < 0.0005 (for details see also supplementary statistical information). (**H**–**O**) Representative confocal images of coronal midbrain sections at 1 month showing DA neurons by immunofluorescence with TH (blue), ACIII (red), and CEP43, as ciliary basal body marker (green). Arrowhead highlights the co-localization of ACIII and CEP43. Scale bar (**A**–**D**): 25 μm; (**E**,**F**,**H**–**O**): 15 μm.

In order to investigate the cell-autonomous role of PC in differentiated DA neurons, we conditionally ablated the *Ift88* gene with the Cre-loxP recombination system. For a spatiotemporal specific deletion of *Ift88*, we employed DAT::Cre transgenic mice, which faithfully express the Cre recombinase in neurons expressing the dopamine transporter (DAT) [28], to generate DAT::Cre;*Ift88*^flox/flox^ mutants (abbreviated: *Ift88* cKO). The DAT::Cre transgene is expressed from E13.5 [28], therefore it is suitable to achieve loss of PC in differentiated DA neurons at late embryonic (E15.5 and E16.5) and postnatal stages (young 1-month-old and mature adult 6-month-old mice) (Figure 1). Immunofluorescence with a specific anti-IFT88 antibody identified the loss of IFT88 in ventral midbrain DA neurons that were labeled by an antibody recognizing tyrosine hydroxylase (TH), an essential enzyme in the dopamine biosynthetic pathway (Figure 1A,B; [26,27]). To further visualize loss of PC in these neurons, we utilized antibodies recognizing adenylyl cyclase III (ACIII) (Figure 1C–F), commonly used as a marker for the PC axoneme [39,40]. As shown in Figure 1C–F, only the basal body was still visible, confirming that the axoneme of the PC was disrupted in the *Ift88* cKO at 1 month. 

To investigate the onset of loss of PC in DA neurons, we quantified the number of TH positive neurons characterized by a visible ACIII staining at E15.5, E16.5, 1 and 6 months (Figure 1G). In E15.5 TH+ neurons, ACIII antibody did not label an elongated axoneme (Figure S1A,B). ACIII was visible as one or two puncta whose number was slightly reduced in the *Ift88* cKO embryos (Figure 1G and Figure S1A,B). Immunofluorescence with Arl13b, a marker of the ciliary membrane, supported the lack of an axonemal structure in TH positive neurons in control E15.5 embryos, while ventral midbrain TH negative cells showed elongated cilia (Figure S2). From E16.5 on, TH positive neurons showed less ACIII staining in the *Ift88* cKO mutant mice in comparison to control littermates and this decrease remained at later stages (ca. 80% of respective controls) (Figure 1G). To further characterize the ACIII puncta visible in the control and *Ift88* cKO mutant mice (Figure 1D,F), we detected the centrosomal protein CEP43, a distal centriolar marker required for anchoring microtubules [41], that labels the ciliary basal body (Figure 1H–O). Co-immunostaining with ACIII antibodies revealed that ACIII may co-localize with this ciliary basal body marker, and ACIII dots were adjacent to the basal body marker in the *Ift88* cKO mutant mice, as shown, for example, in Figure 1M (arrowhead).

### 3.2. Loss of Primary Cilia in Differentiated DA Neurons Reduces Nigrostriatal DA Projections and Striatal Dopamine Content

**Figure 2:**
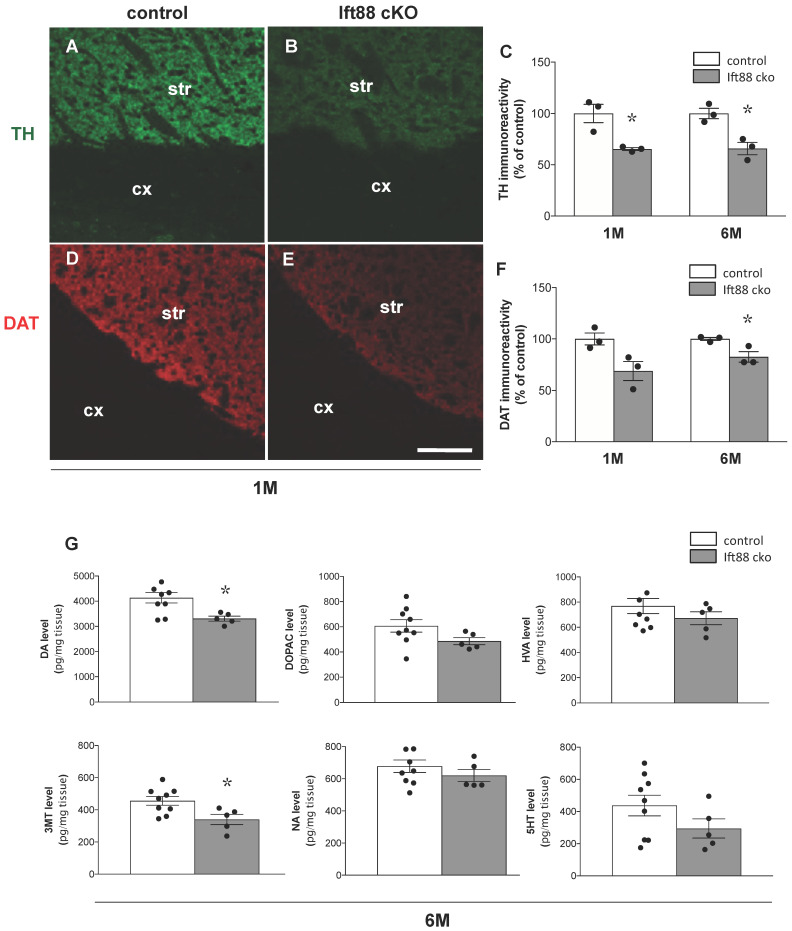
**Loss of primary cilia in DA neurons results in decreased striatonigral integrity.** (**A**,**B**). Representative confocal images of coronal striatal sections showing TH immunoreactivity (green) and (**D**,**E**) DAT immunoreactivity (red) in control and *Ift88* cKO mice at 1 month. (**C**,**F**) Analysis of TH and DAT fluorescence mean intensities in control and *Ift88* cKO dorsal striatum at 1 and 6 months (*n* = 3 mice per group). (**G**) Quantification of dopamine (in figure abbreviated as DA for simplicity), its major metabolites (DOPAC, HVA, 3-MT), and other monoamines (NA, 5-HT) in the striatum of control and *Ift88* cKO mice by HPLC-EC at 6 months (*n* = 9 controls, *n* = 5 *Ift88* cKO). The data are presented as the mean ± SEM. Statistical significance between the indicated groups determined by unpaired two-tailed Student’s *t*-test, * *p* < 0.05 (for details, see also supplementary statistical information). Abbreviations: str, striatum; cx, cortex. Scale bar, 100 μm.

To quantitatively study the extent of the alteration of DA neurons in the *Ift88* cKO mice, we combined immunohistochemical, neurochemical, and stereological analyses. First, we investigated the integrity of the nigrostriatal projections in the *Ift88* cKO mutants, and sections of striatum from *Ift88* cKO mutants and control littermates were stained at 1 and 6 months with two antibodies against TH and DAT (Figure 2A–F). Analysis of immunofluorescence mean intensity in the dorsal striatum revealed a decreased immunoreactivity for both markers (ca. 35% for TH and ca. 20% for DAT at 6 months) (Figure 2C,F), while a similar analysis performed at E16.5 revealed no differences between control and mutant mice (Figure S1C–E). The results were confirmed by Western blot analysis at 1 month, as shown in Figure S3.

Next, we analyzed dopamine levels in the striatum of control and *Ift88* cKO mutants at 6 months (Figure 2G). We found that loss of PC in DA neurons has a long-term impact on the dopamine content in the striatum as well. HPLC-EC assay showed that 6-month-old *Ift88* cKO mutants were characterized by decreased levels of dopamine in the striatum (Figure 2G). No statistically significant differences were observed in dopamine turnover (DOPAC/dopamine, HVA/dopamine; data not shown) and in most of dopamine metabolites, however, we observed decreased levels of 3-MT (Figure 2G).

### 3.3. Loss of Primary Cilia in Differentiated DA Neurons Reduces the Number of Dopamine-Excited SN Neurons

**Figure 3:**
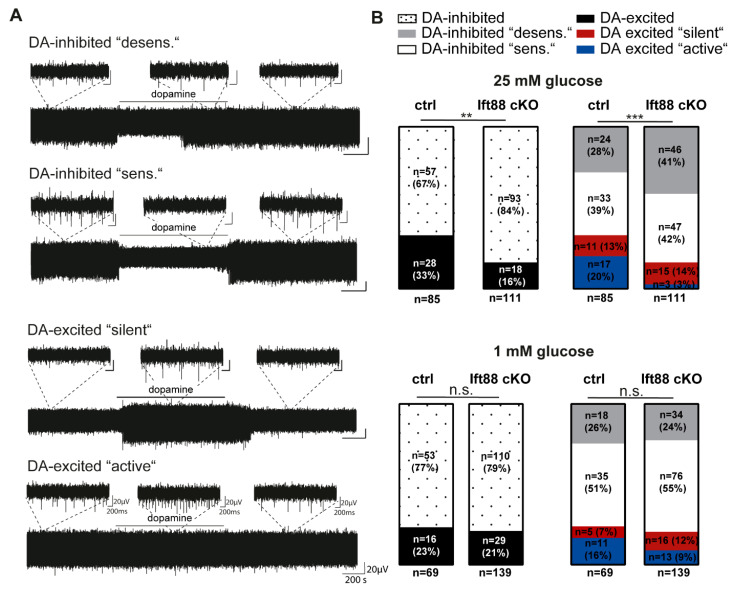
**More SN neurons with inhibitory dopamine responses in *Ift88* cKO mice**. Spontaneous activities and responses to dopamine (in the figure abbreviated as DA, 100 µM, bath applied for 15 min) of SN neurons in brain slices from adult controls and *Ift88* cKO mice, recorded with multi-electrode array techniques (MEA) in ACSF, containing either 25 mM or 1 mM glucose, as indicated. (**A**) Exemplary traces of the four different types of dopamine responses of SN neurons, as described [5]. Inserts display enlarged traces of two seconds. (**B**) SN neurons were classified according to their dopamine responses in dopamine-inhibited cells (inhibited pacemaker activity in dopamine), and dopamine-excited cells (increased pacemaker frequency in dopamine) Dopamine-inhibited cells were subdivided in neurons with (“desens.”, grey) and without (“sens.”, white) prominent desensitization of dopamine responses over time. Dopamine-excited cells were subclassified in neurons without (“silent”, red) and with (“active”, blue) spontaneous activity before dopamine application. In saturated glucose (25 mM), *Ift88* cKO mice displayed significantly more SN neurons with inhibitory dopamine responses (**, *p* = 0.0062), compared to controls (ctrl.), due to significantly lower numbers of dopamine-excited neurons of the “active” type (***, *p* = 0.0001). Statistical comparisons: chi-square tests. Number of analyzed neurons indicated by (*n*). All values and full statistics are detailed in the supplementary statistical information and Supplementary Table S4.

To examine possible functional differences in the *Ift88* cKO mutants, we compared the electrophysiological properties of highly vulnerable SN DA neurons in vital ex vivo brain slice preparations of adult controls and *Ift88* cKO mice (Figure 3, Table S4). We analyzed the typical pacemaker activity (frequency and precision), as well as the response to dopamine (100 µM, bath applied for 15 min) with unbiased extracellular multi-electrode array (MEA) recordings, under metabolic control conditions (ACSF with saturated glucose) and under metabolic stress, induced by glucose deprivation (1 mM glucose). An inhibition of spontaneous activity in response to dopamine (either sustained or with prominent desensitization) has been described as typical for SN DA neurons, and it is mediated by dopamine autoreceptors of the D2 type [42]. However, we and others have described another type of SN neuron that is excited by dopamine, in similar abundance [5,43,44]. These dopamine-excited cells were either silent in the absence of dopamine, or they were active, with a similar pacemaker frequency as the dopamine-inhibited SN DA neurons (Figure 3A, Table S4, and [5]). Surprisingly, while pacemaker activities and inhibitory dopamine response were similar in controls and *Ift88* cKO mice, the number of dopamine-excited SN neurons was significantly reduced in mice without PC in DA neurons (Figure 3B). The lower relative abundance of dopamine-excited SN neurons was in particular due to a significantly reduced amount of the SN subtype that was already active under control conditions (~75% reduction). In addition, the dopamine-excited neurons of the “silent” type from *Ift88* cKO mice displayed significantly lower activities (~50%) in dopamine, in comparison to that of controls (Table S4). These differences were only present in metabolic control conditions (25 mM glucose), but not under metabolic stress (1 mM glucose). 

### 3.4. Loss of Primary Cilia in Differentiated DA Neurons of Ift88 cKO Mutants Renders Them Insensitive to the Neurotoxin MPTP

**Figure 4:**
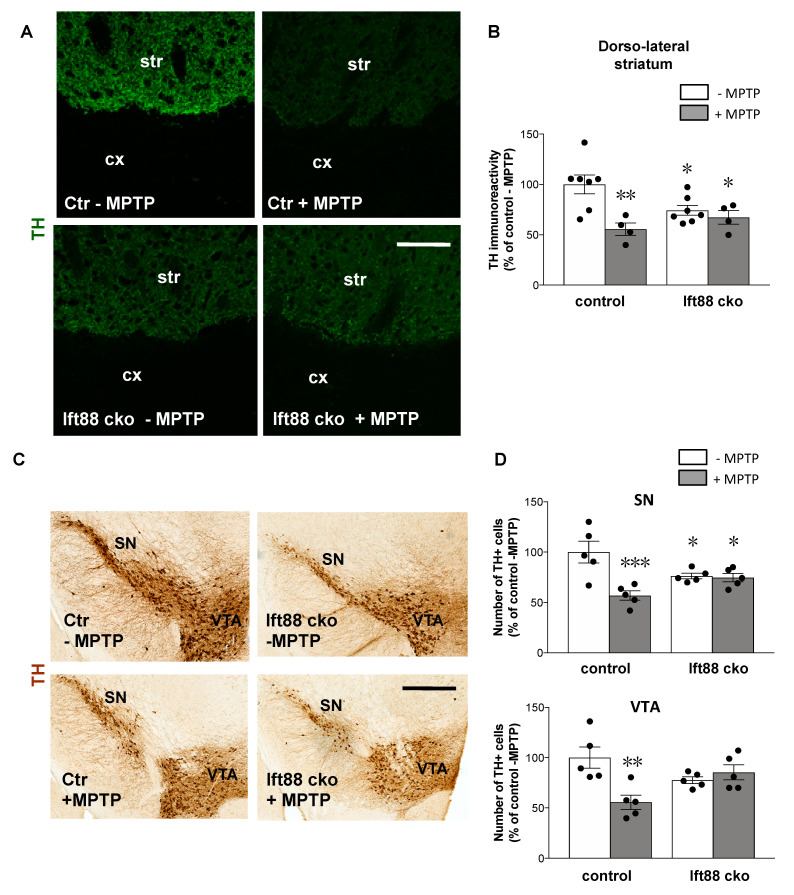
**Loss of primary cilia in DA neurons results in decreased neurotoxic response to MPTP treatment.** (**A**) Representative confocal images of TH immunoreactivity (green) in the dorsal striatum of control and *Ift88* cKO mice with and without MPTP treatment at 6 months. (**B**) Semi-quantitative analysis of TH fluorescence mean intensities in the dorsal striatum of vehicle-treated control and *Ift88* cKO mice (*n* = 7 per group) and of MPTP-treated control and Ift88 cKO mice (*n* = 4 per group). (**C**,**D**) Stereological analysis of the number of DA neurons in the SN and VTA of control and Ift88 cKO mice treated and not treated with MPTP (*n* = 5 per group). The data are presented as the mean ± SEM. Statistical significance between the indicated groups determined by two-way ANOVA followed by Fisher’s LSD test * *p* < 0.05, ** *p* < 0.005, *** *p* < 0.0005 (for details, see also supplementary statistical information). Abbreviations: Ctr, control; str, striatum; cx, cortex. Scale bar, 100 μm (**A**), 500 μm.

Next, we investigated whether maintenance of PC serves a neuroprotective function in ventral midbrain DA neurons. To this end, control and *Ift88* cKO mutant mice were injected intraperitoneally over three days either with vehicle or with MPTP (1-methyl-4-phenyl-1,2,3,6-tetrahydropyridine). The impact on nigrostriatal integrity was analyzed by TH immunoreactivity one day after the last injection (Figure 4A). In control mice, this subacute MPTP treatment resulted in a 45% decrease in the TH positive innervation of the dorsal striatum (Figure 4B). In *Ift88* cKO mutant mice lacking cilia in DA neurons, MPTP treatment did not lead to a further decrease in TH immunoreactivity in the dorsal striatum (Figure 4B). To further validate these findings, we analyzed the number of SN and VTA DA neurons by unbiased stereology in the same mice (Figure 4C,D). The number of DA neurons is reduced upon treatment with MPTP in control mice. Interestingly, the number of TH positive SN DA neurons in the *Ift88* cKO was decreased (ca. 20% fewer DA neurons) in saline-treated animals in comparison to controls (Figure 4D). However, we found a similar number of SN and VTA DA neurons between *Ift88* cKO treated and not treated with MPTP, suggesting that the absence of PC in differentiated DA neurons negatively affected the survival of a subpopulation of SN DA neurons, and rendered the remaining DA neurons insensitive to the effects of MPTP. 

### 3.5. Loss of Primary Cilia in DA Neurons Results in Increased Primary Cilia Length in the Striatum of Ift88 Conditional Knock-Out Mice

**Figure 5:**
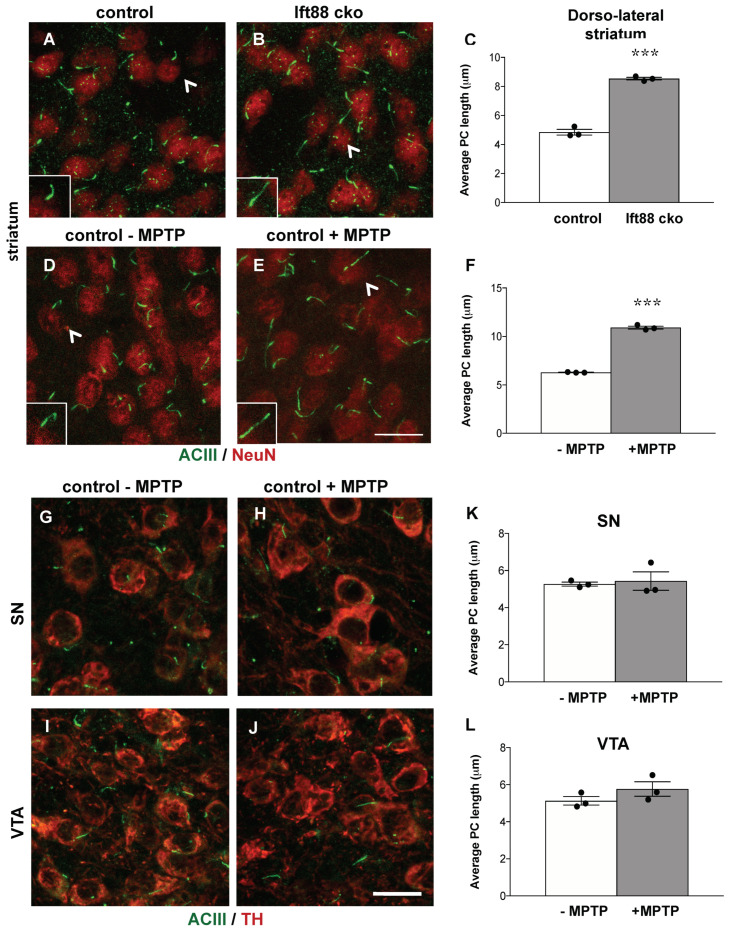
**Loss of striatonigral integrity in the *Ift88* cKO and in the MPTP models is associated with increased length of PC in striatal neurons.** Representative confocal images of dorso-lateral striatal cryosections showing PC immunostained with an antibody recognizing ACIII (green) in NeuN positive neuronal nuclei (red) in control and Ift88 cKO mice (**A**,**B**) and in MPTP-treated and not treated controls (**D**,**E**). Quantification of the average PC length in dorso-lateral striatum in control and *IftI88* cKO mice (**C**) and in control mice upon MPTP treatment (**F**). Arrows point to the PC shown in the inset. (**G**–**J**) Representative confocal images of ventral midbrain cryosections showing PC stained with an antibody recognizing ACIII (green) in TH positive neurons (red) of the SN and VTA in control mice non-treated and treated with MPTP. (**K**,**L**) Quantification of the average PC length in control mice upon MPTP treatment in SN and VTA. At least 80 cilia from each mouse were measured (*n* = 3 mice per group). The data are presented as the mean ± SEM. Statistical significance between the indicated groups determined by unpaired two-tailed Student’s *t*-test, *** *p* < 0.001 (for details, see also supplementary statistical information). Scale bar: (**A**,**B**,**D**,**E**,**G**–**J**); insets: 12 μm.

To establish whether PC in striatal neurons respond to DA input deficits by changes in their length in the Ift88 cKO mice, we examined the length of PC in dorsal striatal neurons, using antibodies against NeuN to label neuronal somata and against ACIII to label primary cilia axoneme (Figure 5A–C). Despite the relatively modest decrease observed in the number of DA neurons and striatal dopamine level in the Ift88 cKO mice (Figure 4), the length of the PC significantly increased in the striatal neurons of the mutants (Figure 5C), almost doubling in their average length (Figure 5C). 

**Figure 6:**
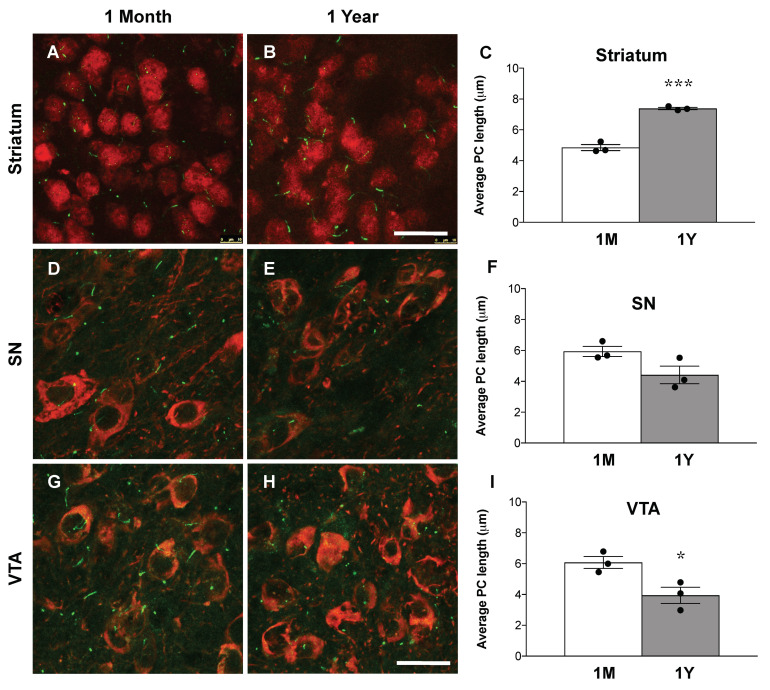
**The length of PC increases during aging in striatal neurons of wildtype mice, but not in DA neurons.** (**A**,**B**,**D**–**H**) Representative confocal images of coronal sections showing ACIII in striatal and SN and VTA DA neurons by immunofluorescence with an ACIII-specific antibody (green) and a TH-specific antibody (red) at 1 month and at 1 year. Quantification of the average PC length in striatal neurons of control mice (**C**) and in SN and VTA DA neurons (**F**,**I**) at 1 month and 1 year. At least 80 cilia from each mouse were measured (*n* = 3 per group). The data are presented as the mean ± SEM. Statistical significance between the indicated groups was determined by unpaired two-tailed Student’s *t*-test; * *p* < 0.05, *** *p* < 0.0005 (for details, see also supplementary statistical information). Scale bars: (**A**,**C**): 25 μm; (**B**,**D**): 50 μm.

Given the impact of the loss of nigrostriatal projections on striatal cilia length upon DA lesions in *Ift88* cKO mutants, we examined the length of PC in dorsal striatal neurons of control mice upon MPTP treatment, using the same approach (Figure 5D–F). Additionally, in this pharmacological model, the length of PC was significantly longer (about 70%) in striatal neurons of MPTP-treated control mice (Figure 5F). Next, we performed the same analysis in SN and VTA DA neurons in the same mice treated and those not treated with MPTP (Figure 5G–L). We found that SN DA and VTA DA neurons show a similar length of primary cilia upon MPTP treatment in control mice (Figure 5K,L), suggesting cell-specific neuroadaptive responses in the striatal neurons caused by DA innervation deficits. We conclude that the increased length of PC in striatal neurons is a response to perturbed nigrostriatal projections shared by different genetic and pharmacological models of DA toxicity. We extended this analysis to young and old control mice (Figure 6). Interestingly, the length of PC in striatal neurons was longer in one-year-old control mice in comparison with one-month-old mice (Figure 6). In SN DA neurons, there were no differences in the length of primary cilia between young and old controls, however, VTA DA neurons revealed a decreased length in one-year-old mice in comparison to one-month-old mice (Figure 6). 

## 4. Discussion

**Figure 7:**
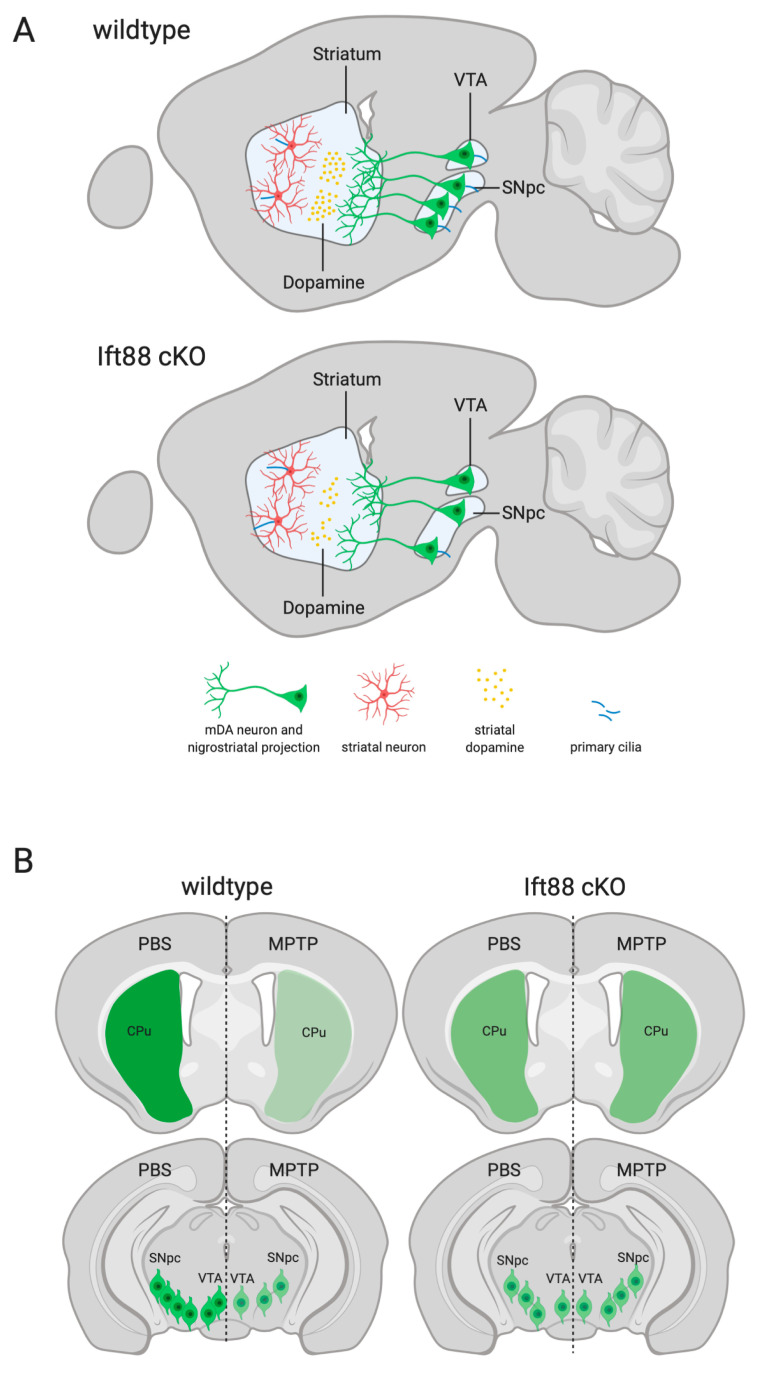
**Summary of the effects of PC loss in DA neurons on striatonigral integrity.** (**A**) In control mice, DA and striatal neurons are characterized by PC (in blue) and dopamine is released in the striatum (yellow circles) via DA projections. In the *Ift88* cKO mice, the number of DA neurons showing primary cilia is drastically reduced, resulting in striatal neurons characterized by an increased length of primary cilia. (**B**) TH immunoreactivity (green) in the striatum and in SN DA neurons is reduced upon MPTP treatment and in the non-treated *Ift88* cKO mice, but this is not exacerbated in the MPTP-treated *Ift88* cKO mice. Abbreviations: Cpu, caudate putamen; SNpc, substantia nigra pars compacta.

In this study, we show that PC are required for the maintenance of SN DA neurons and for nigrostriatal integrity. Our findings suggest that a subpopulation of SN DA neurons is vulnerable to the loss of PC-dependent signaling. Dopamine-excited “active” SN neurons are less common upon loss of PC, while the relative amount of dopamine-inhibited “desensitized” SN DA neurons are increased. These functional differences are only present in metabolic control conditions, but not under metabolic stress, induced by a low level of extracellular glucose.Importantly, MPTP treatment does not impair the survival of the remaining DA neurons, most of which lack PC. Moreover, decreased dopamine as a result of DA PC loss is associated with the elongation of PC in the striatum, suggesting PD-dependent compensatory mechanisms adopted by dopaminoceptive neurons in response to DA lesions, as schematically depicted in Figure 7.

Previous studies in the 6-hydroxydopamine (6-OHDA) neurotoxin rat model and in dopamine D2 (D2R) knock-out mice are consistent with our findings [17,45,46,47]. They have suggested that the elongation of neuronal PC in the dorsal striatum following loss of SN DA neurons is attributable to a lack of stimulation of their postsynaptic D2 receptors [17]. We conclude that increased length of PC in the striatum is a conserved consequence of a reduced DA input. This interpretation is supported by the observation that MPTP treatment does not affect PC length in DA neurons, but it results in ciliary elongation in dopaminoceptive striatal neurons. We also found that length of PC increases in striatal neurons during aging, further suggesting their emerging function in neuroadaptive responses [48]. A similar cell-specific and age-dependent increase in PC length was described in different areas of the hippocampus in wildtype mice [49]. It has been hypothesized that longer cilia tend to be more sensitive in chemo- or mechano-sensation [50,51]. The surface of PC is occupied, among others, by excitatory D1Rs [9]. D1Rs are delivered to cilia from the extraciliary plasma membrane by a mechanism requiring the intraflagellar transport complex-B (IFT-B) [9,10,52]. One could speculate that increased PC length might result in increased D1R ciliary localization, thereby facilitating DA transmission at the postsynaptic level, as a compensatory response to a decrease in DA presynaptic contacts. This mechanism could potentially participate in the neuroadaptation occurring upon DA atrophy in response to drugs of abuse, but also in preclinical stages of PD. Interestingly, a genetic mouse model of Niemann–Pick disease showing a reduced expression of DAT in the striatum also shows subtle changes in the number of PC and high variability in their length [21]. 

The observation that DA neurons lacking PC are more resilient to neurotoxic insults targeting mitochondrial function is an apparent discrepancy, with a recent work showing that the loss of PC results in high vulnerability of DA neurons to MPTP treatment [22]. Differently from this recent study, based on an inducible *Ift88* knock-down approach, we have investigated the cell-autonomous role of PC in a conditional knock-out mouse in which primary cilia are lost during embryonic development only in DA neurons. Moreover, we used a sub-chronic MPTP model with three consecutive injections (at 24 h intervals), and the analysis of the outcome is carried out one day after the last injection. In this application scheme of MPTP, neuronal loss of TH expression and midbrain DA neurodegeneration are highest at early stages, while TH expression later recovers in mice. Thus, analysis after one day is likely to show the peak of neurodegeneration. Previous work has shown that DAT homozygous knock-out mice do not show any significant loss of TH immunoreactivity in the striatum [53,54]. We cannot rule out that reduced DAT expression observed in the *Ift88* conditional knock-out mice lacking PC in DA neurons renders them insensitive to MPTP treatment.

One of the most rigorously investigated functions of PC is their necessity for the transduction of the “canonical” Sonic hedgehog (Shh) pathway. It has been previously shown that genetic ablation of Shh in differentiated DA neurons results in their degeneration, reaching 40% at eight months of age [11]. The conditional knock-out mice of the Shh receptor, *Smoothened*, do not show any impairment of neuronal survival under physiological conditions or under neurotoxic insult [55]. PC-mediated signaling is in particular required in a critical developmental time- indow with a dramatic phenotype when *Ift88* is ablated in midbrain DA progenitors [26,27].

We detected a lower number by about 20% of TH positive SN DA neurons in mice lacking DA PC. We currently do not know when these neurons are lost or de-differentiate or the underlying mechanisms of this decrease. Strikingly, our functional MEA analysis suggests a functional loss of a subpopulation of SN neurons that are excited by dopamine (to a frequency over 10 Hz, i.e., near the maximal frequency of SN DA neurons in ex vivo slices [56]), and that are already active in the absence of dopamine. These neurons should be particularly vulnerable to metabolic stress and degeneration, given their high level of intrinsic metabolic stress [57,58]. However, further studies are required to establish whether loss of PC affects a specific subpopulation of DA neuron migration and terminal differentiation. More specific agents targeting dopamine receptors are required to more precisely address the mechanisms altered by PC loss on DA neurons. We found that PC are not maintained in migrating DA neurons at E15.5. Our data also show that during embryonic development, Arl13b is not expressed in DA neurons, in line with the loss of an ACIII positive axoneme. In neocortical neurons, a developmental lack of axonemal organization (procilium) has been reported, and cilia are not present in migrating neurons in the cortical plate [59].

What could be the physiological role of PC in SN DA neurons? In wildtype mice, we detected a larger number of “active” dopamine-excited SN neurons, and a smaller number of dopamine-inhibited neurons, compared to those of the mutant mice, specifically in metabolic control conditions. We currently do not know much about the physiological function of dopamine-excited SN neurons, nor about the underlying molecular mechanism. We are currently addressing the mechanisms by using specific dopamine receptor agonists and antagonists. With our MEA approach, we could neither identify the projections of dopamine-excited cells, nor whether they were DA or non-DA—or even neurons [5]. However, dopamine-excited SN-derived cultured cells in [44] displayed a positive fluorescent TH-GFP signal, indicating these cells were indeed DA neurons—or at least TH positive. In any case, it is valid to assume that the neurotransmitter release of dopamine-excited SN cells is higher, compared to that of dopamine-inhibited SN DA neurons, and that they also possess a higher level of intrinsic metabolic stress, due to their demanding electrical activity that is further stimulated by dopamine.

The primary cilium constitutes an electrophysiological micro-domain in neurons [20,60,61]. In highly vulnerable SN DA neurons, the PC might sense the metabolic state of the organism, e.g., by sensing extracellular glucose levels, as has been described for PC of pancreatic beta-cells [4], or by sensing intracellular calcium levels [62]. They might be important to adapt the ratio of dopamine-excited and dopamine-inhibited SN neurons to the current metabolic state. In line with this, it has been shown that mitochondrial stress-induced ciliogenesis is mediated by mitochondrial reactive oxygen species generation, subsequent activation of AMP-activated protein kinase, and autophagy [22]. These factors are all known stressors for neurodegeneration and PD [18]. 

Future studies are needed to investigate if and how alterations in PC contribute to metabolic sensing of DA neurons and to neurodegeneration in PD patients. Although PD is not classified as a ciliopathy, subtle changes in primary cilia-dependent signaling may have pathological consequences. 

We provide new evidence for a dynamic crosstalk between dopamine and PC, and for their role in neuroadaptation. In conclusion, this study offers a new model to better understand the role of PC in the dopamine system in health and disease. 

## Data Availability

Data is contained within the article.

## Supplementary Materials

The following are available online at https://www.mdpi.com/article/10.3390/antiox10081284/s1, Figure S1: Analysis of ventral midbrain DA neurons showing PC and of TH striatal immunoreactivity at embryonic stages. Figure S2: Arl13b does not label primary cilia in TH immunostained neurons at E15.5. Figure S3: Analysis of TH and DAT expression in dorsal striatum by Western blots. Table S1–S13: Supplementary statistical information.
