# Supplementary material for: Targeted Ablation of Primary Cilia in Differentiated Dopaminergic Neurons Reduces Striatal Dopamine and Responsiveness to Metabolic Stress
Source: Antioxidants (Basel). 2021 Aug 13;10(8):1284. doi: 10.3390/antiox10081284 (PMC8389284; doi:10.3390/antiox10081284)
Supplement: Supplementary file 1 [file antioxidants-10-01284-s001.zip › antioxidants-1312973-supplementary.pdf]

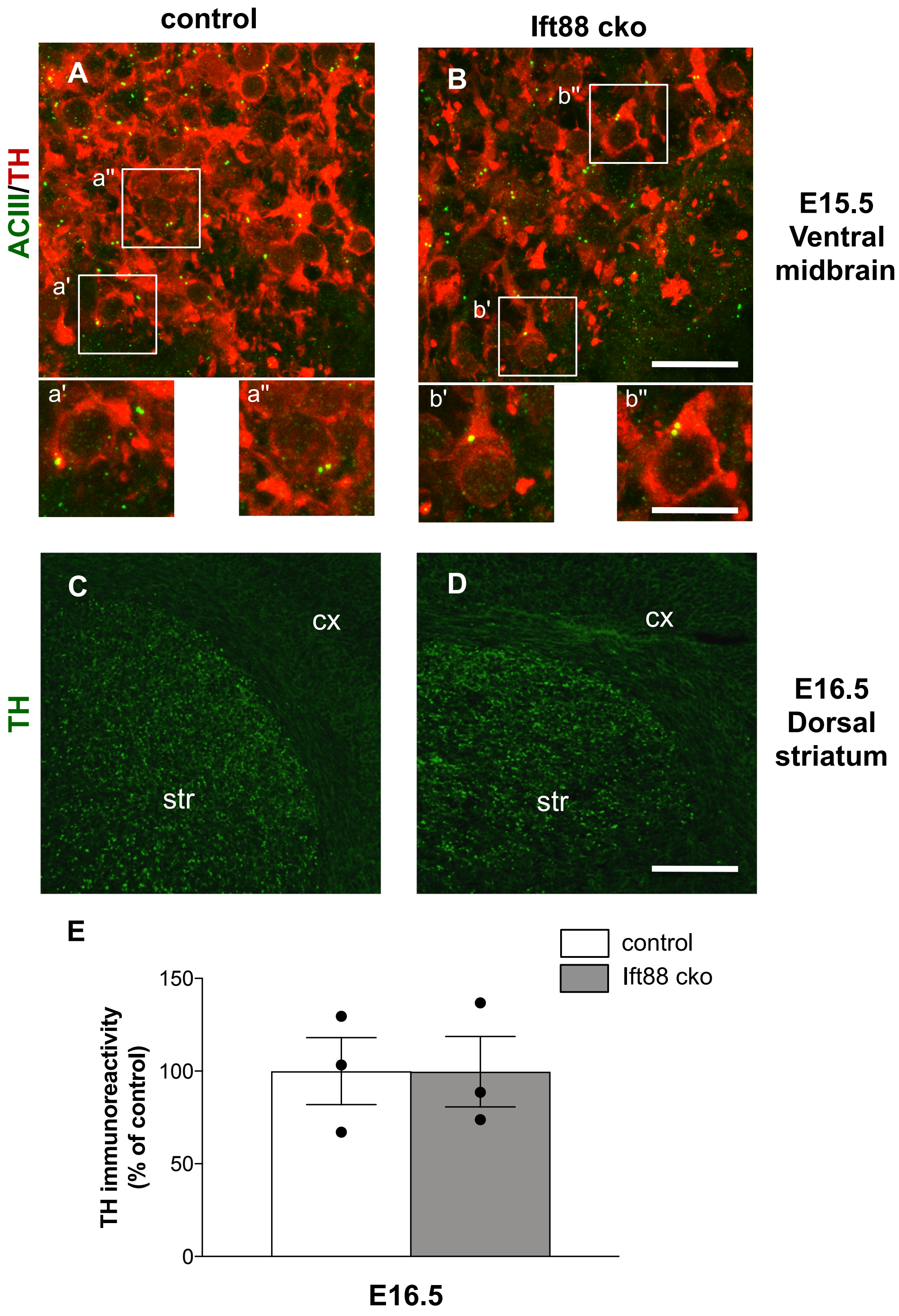

Figure S1: Analysis of primary cilia in ventral midbrain dopaminergic neurons and of TH striatal immunoreactivity at embryonic stages. (A,B) Representative confocal images of coronal midbrain sections at E15.5 by immunofluorescence with an ACIII-specific antibody (green) and a TH-specific antibody (red) in control and *Ift88* cKO. Insets (a', a'', b', b'') show higher magnification of the boxed areas in panels A and B, respectively. (C,D) Representative confocal images of coronal striatal sections showing TH immunoreactivity (green) at E16.5 in control and *Ift88* cKO. (E) Semi-quantitative analysis of TH immunoreactivity in the dorso-lateral striatum in control and *Ift88* cKO mutant littermates at E16.5. The data are presented as the mean ±SEM. No statistical significance between the indicated groups was determined by unpaired two-tailed Student's t test (for details see also Supplementary statistical information). Scale bars: A,B: 20 µm, insets: 10 µm, and C,D: 100 µm. Abbreviations: str, striatum; cx, cortex.

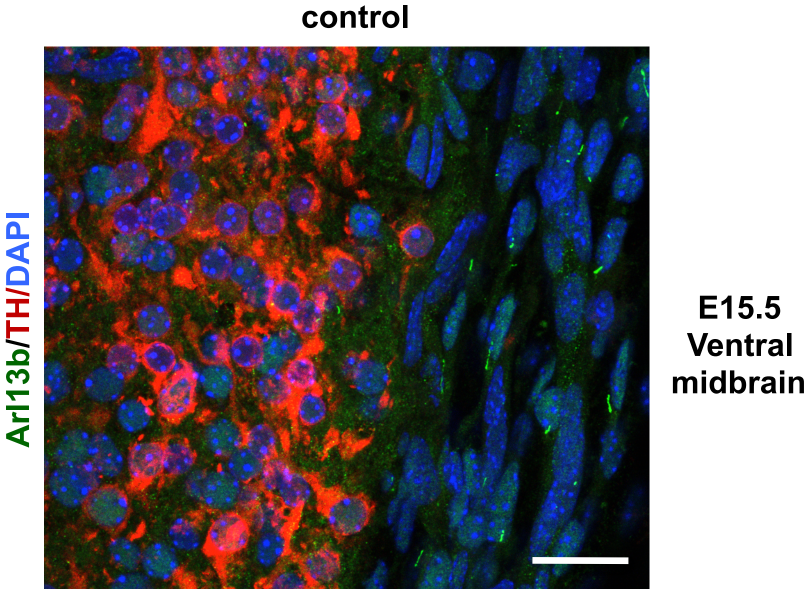

Figure S2: Arl13b does not label primary cilia in TH immunostained neurons at E15.5. Representative confocal image of coronal midbrain sections showing PC labeled by immunofluorescence with an Arl13b-specific antibody (green) in TH negative cells identified by nuclear DAPI staining (blue). A TH-specific antibody (red) is used to identify DA neurons in a E 15.5 control embryo. Scale bar: 20 µm.

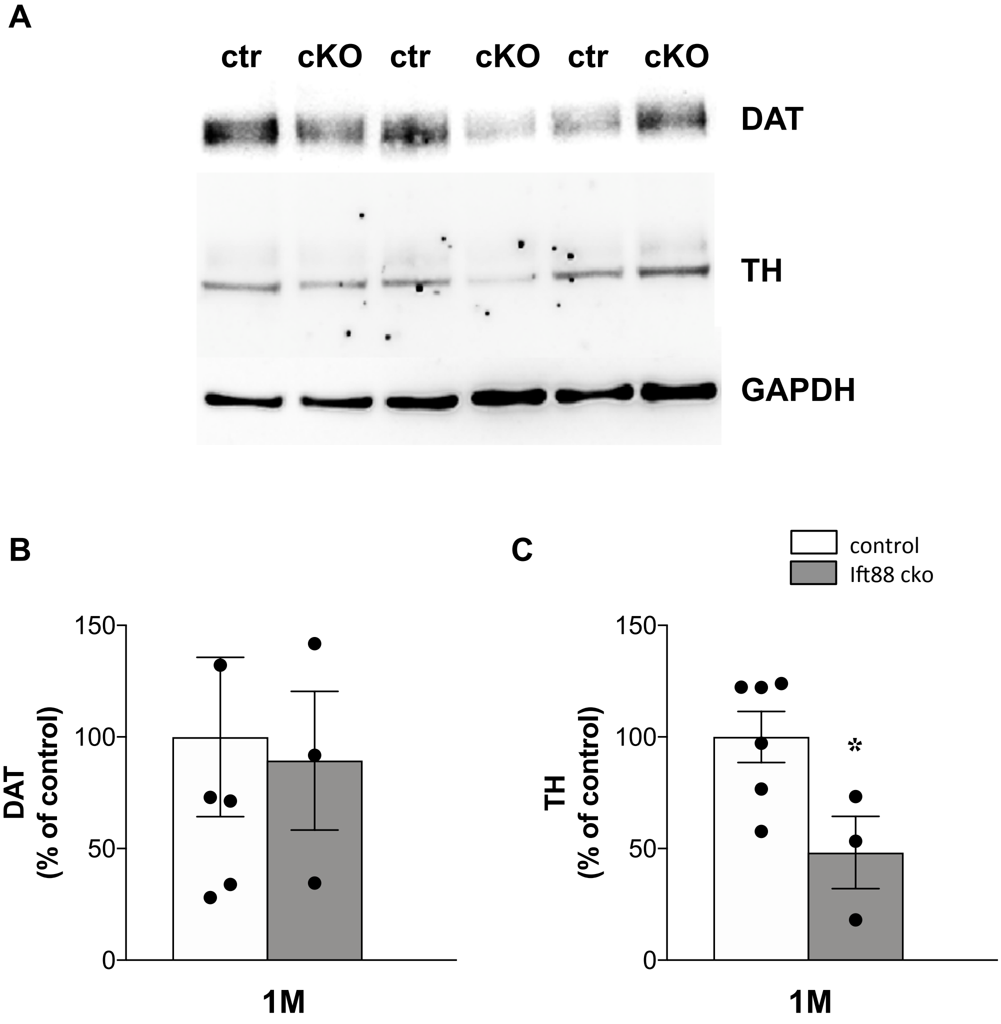

Figure S3: Analysis of TH and DAT expression in dorsal striatum by Western blots. (A) Western blot analysis of DAT, TH and GAPDH in dorsal striatum of control and *Ift88* cKO mice. (B, C) Densitometric quantification of DAT and TH protein level expressed as percentage of controls. The data are presented as the mean ±SEM. Statistical significance between the indicated groups was determined by unpaired two-tailed Student's t test (for details see also Supplementary statistical information). Abbreviations: ctr: control, M, month.

Supplementary statistical information

Suppl. Table S1

Data and statistics for graphs shown in **Fig. 1G**, n: total number of analysed TH positive neurons, N: number of mice. Statistical significance is shown according to two-way ANOVA.

**G) TH+ cells with the PC (%)**						
	**Mean**	**±SD**	**±SEM**	**Median**	**95% CI**	**n/N**
**E15.5 control**	91,97	3,75	2,16	92.04	82.66 – 101.3	663/3
**E15.5 Ift88^{DatCre}**	83,92	5,49	3,17	84.48	70.29 – 97.56	720/3
**E16.5 control**	91,38	3,10	1,79	90.65	83.67 – 99.09	728 /3
**E16.5 Ift88^{DatCre}**	38,48	4,38	2,53	38.01	27.60 – 49.36	535/3
**1 mo control**	98,04	1,66	0,96	98.08	93.92 – 102.2	848/3
**1 mo Ift88^{DatCre}**	25,11	5,71	3,29	23.73	10.92 – 39.31	720/3
**6 mo control**	97,42	1,25	0,72	97.46	94.33 – 100.5	582/3
**6 mo Ift88^{DatCre}**	22,03	0,88	0,51	21.87	19.85 – 24.22	771/3

***Two-way ANOVA**	
**Source of Variation**	**P value**
**Interaction**	<0.0001
**Age**	<0.0001
**Genotype**	<0.0001

**Results of Fisher's LSD multiple comparison**	**Summary**	**P value**
**E15.5 control vs. E15.5 Ift88^{DatCre}**	*	0.0175
**E15.5 control vs. E16.5 Ift88^{DatCre}**	****	<0.0001
**E15.5 control vs. 1 mo Ift88^{DatCre}**	****	<0.0001
**E15.5 control vs. 6 mo Ift88^{DatCre}**	****	<0.0001
**E16.5 control vs. E15.5 Ift88^{DatCre}**	*	0.0259
**E16.5 control vs. E16.5 Ift88^{DatCre}**	****	<0.0001
**E16.5 control vs. 1mo Ift88^{DatCre}**	*	0.0434
**E16.5 control vs. 6 mo Ift88^{DatCre}**	****	<0.0001
**1 mo control vs. E15.5 Ift88^{DatCre}**	***	0.0003
**1 mo control vs. E16.5 Ift88^{DatCre}**	****	<0.0001
**1 mo control vs. 1 mo Ift88^{DatCre}**	****	<0.0001
**1 mo control vs. 6 mo Ift88^{DatCre}**	****	<0.0001
**6 mo control vs. E15.5 Ift88^{DatCre}**	***	0.0004
**6 mo control vs. E16.5 Ift88^{DatCre}**	****	<0.0001
**6 mo control vs. 1mo Ift88^{DatCre}**	****	<0.0001
**6 mo control vs. 6 mo Ift88^{DatCre}**	****	<0.0001

Suppl. Table S2

Data and statistics for graphs shown in **Fig. 2C**, n: number of analysed striatal sections per mouse, N: number of mice. Statistical significance is shown according to two-tailed unpaired t-test.

2C) TH immunoreactivity (% of relative control)						
	**Mean**	**±SD**	**±SEM**	**Median**	**95% CI**	**n/N**
**1 mo control**	100	15.57	8.99	106.9	61.33 – 138.7	7/3
**1 mo Ift88^{DatCre}**	65.32	2.38	1.38	65.53	59.41 – 71.24	7/3
**6 mo control**	100	8.86	5.12	98.70	77.98 – 122.0	7/3
**6 mo Ift88^{DatCre}**	65.84	10.44	6.02	67.87	39.91 – 91.76	7/3

Two-tailed unpaired t-test	Summary	P value
**1 mo control vs. 1 mo Ift88^{DatCre}**	*	0.019
**6 mo control vs. 16mo Ift88^{DatCre}**	*	0.012

Data and statistics for graphs shown in **Fig. 2F**, n: number of analysed striatal sections, N: number of mice. Statistical significance is shown according to two-tailed unpaired t-test.

2F) DAT immunoreactivity (% of relative control)						
	**Mean**	**±SD**	**±SEM**	**Median**	**95% CI**	**n/N**
**1 mo control**	100	10.13	5.85	97.41	74.84 – 125.2	7/3
**1 mo Ift88^{DatCre}**	68.96	15.97	9.22	73.43	29.30 – 108.6	7/3
**6 mo control**	100	2.58	1.49	101.2	93.59 – 106.4	7/3
**6 mo Ift88^{DatCre}**	82.72	8.90	5.14	77.6	60.62 – 104.8	7/3

Two-tailed unpaired t-test	Summary	P value
**1 mo control vs. 1 mo Ift88^{DatCre}**	*	0.047
**6 mo control vs. 6mo Ift88^{DatCre}**	*	0.032

Suppl. Table S3

Data and statistics for graphs shown in **Fig. 2G**, N: number of mice. Statistical significance is shown according to two-tailed unpaired t-test.

	2G) HPLC analysis of DA, DOPAC, HVA, 3MT, NA and 5HT (pg/mg tissue)						
		**Mean**	**±SD**	**±SEM**	**Median**	**95% CI**	**N**
**Dopamine**	**6 mo control**	4137	615.0	205.0	4273	3664 – 4609	9
	**6 mo Ift88^{DatCre}**	3310	218.9	97.89	3296	3038 - 3582	5
**DOPAC**	**6 mo control**	606.9	147.0	49.02	572.8	493.9 –719.9	9
	**6 mo Ift88^{DatCre}**	486.1	62.4	27.91	461.5	408.6 –563.6	5
**HVA**	**6 mo control**	768.7	180.1	60.03	703.2	630.2 –907.1	9
	**6 mo Ift88^{DatCre}**	671.7	114.6	51.24	718.9	529.4 –814.0	5
**3MT**	**6 mo control**	456.1	80.7	26.9	470.7	394.1 –518.2	9
	**6 mo Ift88^{DatCre}**	340.3	71.4	31.92	367.1	251.6 –428.9	5
**NA**	**6 mo control**	677.5	115.8	38.62	648.3	588.4 –766.5	9
	**6 mo Ift88^{DatCre}**	620.2	83.2	37.22	564.1	516.8 –723.5	5
**5HT**	**6 mo control**	437.4	193.7	64.58	437.4	288.5 –586.3	9
	**6 mo Ift88^{DatCre}**	327.4	129.0	64.48	294.6	128.8 –460.4	5

Two-tailed unpaired t-test	Summary	P value
**Dopamine 6 mo control vs. 6 mo Ift88^{DatCre}**	*	0.01
**DOPAC 6 mo control vs. 6mo Ift88^{DatCre}**	ns	0.11
**HVA 6 mo control vs. 6mo Ift88^{DatCre}**	ns	0.30
**3MT 6 mo control vs. 6mo Ift88^{DatCre}**	*	0.02
**NA 6 mo control vs. 6mo Ift88^{DatCre}**	ns	0.35
**5HT 6 mo control vs. 6mo Ift88^{DatCre}**	ns	0.33

Suppl. Table S4

Pacemaker activities and dopamine-responses of SN neurons from adult control and Ift88 cKO mice. MEA-recordings from **Fig. 3** in ACSF containing 25 mM glucose (upper), and 1 mM glucose (lower), as indicated. Pacemaker frequencies and pacemaker precision are given as mean basal firing rate and % coefficient of variance of the interspike interval (CV-ISI), respectively, during the first 10 minutes of recordings. “Activity at min 15 in dopamine [Hz]” was calculated as mean firing rate during the last minute of dopamine-application (100 µM, 15 min). n represents the number of analyzed cells (in brackets, the relative % of this cell-type), N represents the number of analyzed mice. Data are given as mean ± SEM. Datasets marked by (#) were not normally distributed. Significances/p-values according to unpaired Mann-Whitney tests, and Chi-square tests as indicated for comparison of relative abundances of dopamine-excited vs dopamine-inhibited SN neurons, and of the four different types of dopamine responses. P-values indicating significant differences are given in bold.

**Dopamine responses (25 mM glucose)**	**control mice**				
	**Basal firing rate [Hz]**	**CV-ISI [%]**	**Activity at min 15 in dopamine [Hz]**	**n (%)**	**N**
dopamine-excited, “silent”, no spontaneous activity	0	11.3 ± 1.0	8.5 ± 1.9	11 (13)	3
dopamine-excited, “active”, spontaneous activity	4.4 ± 0.7	6.8 ± 0.9 #	11.9 ± 1.3	17 (20)	3
dopamine-inhibited, sensitized D2-AR	1.76 ± 0.2 #	15.1 ± 1.1	0	33 (39)	3
dopamine-inhibited, desensitized D2-AR	2.3 ± 0.2	15.1 ± 1.4	1.7 ± 0.2	24 (28)	3
	**Ift88 cKO mice**				
dopamine-excited, “silent”, no spontaneous activity	0	12.6 ± 1.1	3.7 ± 0.4	15 (14)	4
dopamine-excited, “active”, spontaneous activity	2.9 ± 0.9	6.0 ± 0.1	12.1 ± 3.8	3 (3)	4
dopamine-inhibited, sensitized D2-AR	1.9 ± 0.1 #	14.8 ± 1.1 #	0	47 (42)	4
dopamine-inhibited, desensitized D2-AR	2.1 ± 0.1	15.2 ± 0.9 #	1.5 ± 0.2 #	46 (41)	4
p-values (controls versus Ift88 cKO)	dopamine-silent: 0.4444 dopamine-	dopamine-silent: 0.5309 dopamine-	dopamine-silent: **0.0401** dopamine-active:	Chi-square: **p = 0.0062**	

	active: 0.5456 dopamine-sens: 0.4777 dopamine-desens: 0.3857	active: 0.8751 dopamine-sens: 0.7558 dopamine-desens: 0.9755	>0.9999 dopamine-sens: >0.9999 dopamine-desens: 0.5683	**p = 0.0001**

**Dopamine responses (1 mM glucose)**	**control mice**				
	**Basal firing rate [Hz]**	**CV-ISI [%]**	**Activity at min 15 in DA [Hz]**	**n (%)**	**N**
dopamine-excited, "silent", no spontaneous activity	0	14.7 ± 3.7	7.6 ± 1.9	5 (7)	4
dopamine -excited, "active", spontaneous activity	6.7 ± 1.5 #	6.1 ± 1.1	14.3 ± 2.4	11 (16)	4
dopamine -inhibited, sensitized D2-AR	1.7 ± 0.1 #	14.0 ± 1.1	0	35 (51)	4
dopamine -inhibited, desensitized D2-AR	1.6 ± 0.2	16.5 ± 1.4	0.89 ± 0.1	18 (26)	4
**Ift88 cKO mice**					
dopamine -excited, "silent", no spontaneous activity	0	10.3 ± 1.4	7.1 ± 1.0	16 (12)	4
dopamine -excited, "active", spontaneous activity	3.7 ± 0.6	7.9 ± 0.9	10.1 ± 1.0 #	13 (9)	4
dopamine -inhibited, sensitized D2-AR	1.5 ± 0.08 #	14.2 ± 0.8 #	0	76 (55)	4
dopamine -inhibited, desensitized D2-AR	1.7 ± 0.1	14.1 ± 1.1	0.8 ± 0.1	34 (24)	4
(controls versus Ift88 cKO)	dopamine-silent: >0.9999 dopamine-active: 0.0635 dopamine-sens: 0.0896 dopamine-desens: 0.6681	dopamine-silent: 0.3097 dopamine-active: 0.192 dopamine-sens: 0.7307 dopamine-desens: 0.2888	dopamine-silent: 0.7241 dopamine-active: 0.1623 dopamine-sens: >0.9999 dopamine-desens: 0.8229	Chi-square: p = 0.7014 p = 0.4319	

Additional p-values/significances within groups assessed with Kruskal Wallis with Dunn´s multiple comparison test.

**Dopamine responses (25 mM glucose)**	**control mice**		
	**Basal firing rate [Hz]**	**CV-ISI [%]**	**Activity at min 15 in DA [Hz]**
Desens vs sens	0.5611	>0.9999	**0.0002**

Desens vs silent	**<0.0001**	0.9274	0.0733
Desens vs acive	>0.9999	**0.0003**	**0.0007**
Sens vs silent	**0.0027**	0.6691	**<0.0001**
Sens vs active	**0.0307**	**<0.0001**	**<0.0001**
Silent vs active	**<0.0001**	0.2539	>0.9999
	**Ift88 cKO mice**		
	**Basal firing rate [Hz]**	**CV-ISI [%]**	**Activity at min 15 in DA [Hz]**
Desens vs sens	>0.9999	>0.9999	**<0.0001**
Desens vs silent	**<0.0001**	0.5592	**0.0564**
Desens vs acive	>0.9999	**0.0054**	0.2278
Sens vs silent	**<0.0001**	0.4188	**<0.0001**
Sens vs active	0.9841	**0.0013**	**<0.0001**
Silent vs active	**0.0014**	>0.9999	>0.9999

**Dopamine responses (1 mM glucose)**	**control mice**		
	**Basal firing rate [Hz]**	**CV-ISI [%]**	**Activity at min 15 in DA [Hz]**
Desens vs sens	>0.9999	>0.9999	**<0.0001**
Desens vs silent	0.6070	>0.9999	0.7797
Desens vs acive	**0.0007**	**0.0002**	**0.0353**
Sens vs silent	**0.0266**	>0.9999	**<0.0001**
Sens vs active	**0.0003**	**0.0030**	**<0.0001**
Silent vs active	**<0.0001**	0.1176	>0.9999
	**Ift88 cKO mice**		
	**Basal firing rate [Hz]**	**CV-ISI [%]**	**Activity at min 15 in DA [Hz]**
Desens vs sens	0.8157	>0.9999	**<0.0001**
Desens vs silent	**<0.0001**	0.5592	0.1420
Desens vs active	0.2677	**0.0054**	**0.0125**
Sens vs silent	**<0.0001**	0.4188	**<0.0001**
Sens vs active	**0.0052**	**0.0013**	**<0.0001**
Silent vs active	**<0.0001**	>0.9999	>0.9999

Suppl. Table S5

Data and statistics for graphs shown in **Fig. 4B**, n: total number of analysed striatal sections, N: number of mice. Statistical significance is shown according to two-way ANOVA.

4B) TH immunoreactivity (% of control w/o MPTP)						
	**Mean**	**±SD**	**±SEM**	**Median**	**95% CI**	**n/N**
**Control w/o MPTP**	100	24,79	9,37	105.3	77.08 – 122.9	7/7
**Control w/ MPTP**	55,41	12,50	6,24	56.18	35.55 – 75.26	7/4
**Mutant w/o MPTP**	74,24	13,11	4,95	69.61	62.12 – 86.37	7/7
**Mutant w/ MPTP**	67,20	13,50	6,75	69.88	45.71 – 88.65	7/4

*Two-way ANOVA	
**Source of Variation**	**P value**
**Interaction**	0.029
**Treatment**	0.0043
**Genotype**	0.388

Results of Fisher's LSD multiple comparison	Summary	p value
**Control w/o MPTP vs. Control w/ MPTP**	***	0.001
**Control w/o MPTP vs. Mutant w/o MPTP**	*	0.015
**Control w/o MPTP vs. Mutant w/ MPTP**	**	0.009
**Mutant w/o MPTP vs. Control w/ MPTP**	ns	0.110
**Mutant w/o MPTP vs. Mutant w/ MPTP**	ns	0.535
**Control w/ MPTP vs. Mutant w/ MPTP**	ns	0.363

Suppl. Table S6

Data and statistics for graphs shown in **Fig. 4D (upper panel)**, N: number of mice. Statistical significance is shown according to two-way ANOVA.

	4D) Number of TH positive neurons in SN (% of control w/o MPTP)						
	**Raw numbers ±SEM**	**Mean**	**±SD**	**±SEM**	**Median**	**95% CI**	**N**
**Control w/o MPTP**	13292,98 ±1443,83	100	24.29	10.86	96.61	69.85 – 130.2	5
**Control w/ MPTP**	7553,68 ±607,4	56.82	10.22	4.57	57.74	44.14 – 69.51	5
**Mutant w/o MPTP**	10132,25 ±370,08	76.22	6.23	2.79	74.22	68.50 – 83.95	5
**Mutant w/ MPTP**	9922,19 ±548,25	74.64	9.22	4.12	74.33	63.19 – 86.09	5

*Two-way ANOVA	
**Source of Variation**	**P value**
**Interaction**	0.005
**Treatment**	0.65
**Genotype**	0.003

Results of Fisher's LSD multiple comparison	Summary	p value
**Control w/o MPTP vs. Control w/ MPTP**	*	0.018
**Control w/o MPTP vs. Mutant w/o MPTP**	***	0.0002
**Control w/o MPTP vs. Mutant w/ MPTP**	*	0.013
**Mutant w/o MPTP vs. Control w/ MPTP**	*	0.048
**Mutant w/o MPTP vs. Mutant w/ MPTP**	ns	0.067
**Control w/ MPTP vs. Mutant w/ MPTP**	ns	0.862

Suppl. Table S7

Data and statistics for graphs shown in **Fig. 4D (lower panel)**, N: number of mice. Statistical significance is shown according to two-way ANOVA.

4D) Number of TH positive neurons in VTA (% of control w/o MPTP)							
	**Raw numbers ±SEM**	**Mean**	**±SD**	**±SEM**	**Median**	**95% CI**	**N**
**Control w/o MPTP**	**16316.28** ±1726.9	100	23.67	10.6	89.36	70.61 – 129.4	5
**Control w/ MPTP**	**9082.73** ±1150.4	55.67	15.77	7.1	56.12	36.09 – 75.24	5
**Mutant w/o MPTP**	**12648.85** ±539.1	77.52	7,.9	3.3	76.77	68.35 – 86.69	5
**Mutant w/ MPTP**	**13929.94** ±1240.1	85.37	16.99	7.6	80.99	64.27 – 106.5	5

*Two-way ANOVA	
**Source of Variation**	**P value**
**Interaction**	0.003
**Treatment**	0.029
**Genotype**	0.641

Results of Fisher's LSD multiple comparison	Summary	P value
**Control w/o MPTP vs. Control w/ MPTP**	***	0.001
**Control w/o MPTP vs. Mutant w/o MPTP**	ns	0.052
**Control w/o MPTP vs. Mutant w/ MPTP**	ns	0.192
**Mutant w/o MPTP vs. Control w/ MPTP**	ns	0.059
**Mutant w/o MPTP vs. Mutant w/ MPTP**	ns	0.475
**Control w/ MPTP vs. Mutant w/ MPTP**	*	0.014

Suppl. Table S8

Data and statistics for graphs shown in **Fig. 5C,F**, n: total number of analysed NeuN positive striatal neurons, N: number of mice. Statistical significance is shown according to two-tailed unpaired t-test.

**5C) Average PC length (µm)**						
	**Mean**	**±SD**	**±SEM**	**Median**	**95% CI**	**n/N**
**1 mo control**	4.85	0.33	0.19	4.686	4.02 – 5.68	240/3
**1 mo Ift88^{DatCre}**	8.54	0.16	0.09	8.533	8.14 – 8.93	240/3

**Two-tailed unpaired t-test**	**Summary**	**P value**
**control vs. Ift88^{DatCre}**	****	<0.0001

**5F) mean PC length (µm)**						
	**Mean**	**±SD**	**±SEM**	**Median**	**95% CI**	**n/N**
**6 mo control w/o MPTP**	6.29	0.04	0.02	6.271	6.20 – 6.38	240/3
**6 mo control w/ MPTP**	10.9	0.25	0.14	10.84	10.28 – 11.52	240/3

**Two-tailed unpaired t-test**	**Summary**	**P value**
**6 mo control w/o MPTP vs. control w/ MPTP**	****	<0.0001

Suppl. Table S9

Data and statistics for graphs shown in **Fig. 5K, L**, n: total number of analysed TH positive neurons in SN, N: number of mice. Statistical significance is shown according to two-tailed unpaired t-test.

5K) mean PC length (μm)						
	**Mean**	**±SD**	**±SEM**	**Median**	**95% CI**	**n/N**
**6 mo control w/o MPTP**	5.27	0.18	0.10	5.242	4.82 – 5.72	240/3
**6 mo control w/ MPTP**	5.43	0.86	0.5	4.958	3.29 – 7.58	240/3

Two-tailed unpaired t-test	Summary	P value
**control w/o MPTP vs. control w/ MPTP**	ns	0.76

5L) mean PC length (μm)						
	**Mean**	**±SD**	**±SEM**	**Median**	**95% CI**	**n/N**
**6 mo control w/o MPTP**	5.13	0.4	0.2	4.991	4.15 – 6.19	240/3
**6 mo control w/ MPTP**	5.77	0.7	0.4	5.596	4.07 – 7.45	240/3

Two-tailed unpaired t-test	Summary	P value
**control w/o MPTP vs. control w/ MPTP**	ns	0.23

Suppl. Table S10

Data and statistics for graphs shown in **Fig. 6C**, n: total number of analysed NeuN positive neurons in the striatum, N: number of mice. Statistical significance is shown according to two-tailed unpaired t-test.

6C) mean PC length (um)						
	**Mean**	**±SD**	**±SEM**	**Median**	**95% CI**	**n/N**
**1 mo control**	4.85	0.33	0.19	4.686	4.02 – 5.68	240/3
**12 mo control**	7.39	0.13	0.07	7.359	7.07 – 7.70	240/3

Two-tailed unpaired t-test	Summary	P value
**control vs. Ift88^{DatCre}**	***	0.0002

Suppl. Table S11

Data and statistics for graphs shown in **Fig. 6F**, n: total number of analysed TH positive neurons in SN and VTA, N: number of mice. Statistical significance is shown according to two-tailed unpaired t-test.

**6F) mean PC length (µm) SN**						
	**Mean**	**±SD**	**±SEM**	**Median**	**95% CI**	**n/N**
**1 mo control**	5.94	0.57	0.33	5.679	4.53 – 7.35	240/3
**12 mo control**	4.42	0.99	0.57	4.098	1.95 – 6.88	240/3

**Two-tailed unpaired t-test**	**Summary**	**p value**
**control vs. Ift88^{DatCre}**	n.s.	**0.08**

**6I) mean PC length (µm) VTA**						
	**Mean**	**±SD**	**±SEM**	**Median**	**95% CI**	**n/N**
**1 mo control**	6.08	0.67	0.39	5.993	4.41 – 7.75	240/3
**12 mo control**	3.95	0.91	0.53	4.073	1.68 – 6.21	240/3

**Two-tailed unpaired t-test**	**Summary**	**P value**
**control vs. Ift88^{DatCre}**	*	0.03

Suppl. Table S12

Data and statistics for graphs shown in **Suppl. Fig. 1**, n: total number of analysed positive striatal sections, N: number of mice. Statistical significance is shown according to two-tailed unpaired t-test.

**TH immunoreactivity (% of control)**						
	**Mean**	**±SD**	**±SEM**	**Median**	**95% CI**	**n/N**
**E16.5 control**	100	31.35	18.1	103.4	22.12 – 177.9	7/3
**E16.5 Ift88^{DatCre}**	99.7	32,93	19.0	88.52	17.89 – 181.5	7/3

**Two-tailed unpaired t-test**	**Summary**	**P value**
**control vs. Ift88^{DatCre}**	n.s.	0.99

Suppl. Table S13

Data and statistics for graphs shown in **Suppl. Fig. 3**, N: number of mice. Statistical significance is shown according to two-tailed unpaired t-test.

DAT densitometric analysis (a.u.) (% of control)						
	**Mean**	**±SD**	**±SEM**	**Median**	**95% CI**	**N**
**control**	100	87.5	35.7	72.11	8.21 – 191.8	6
**Ift88^{DatCre}**	89.4	53.7	31.0	91.75	-44.04 – 222.8	3

**Two-tailed unpaired t-test**	**Summary**	**P value**
**control vs. Ift88^{DatCre}**	n.s	0.855

TH densitometric analysis (a.u.) (% of control)						
	**Mean**	**±SD**	**±SEM**	**Median**	**95% CI**	**N**
**control**	100	27.9	11.4	109.7	70.69 - 129.3	6
**Ift88^{DatCre}**	48.2	28	16.2	53.36	-21.23 – 117.8	3

**Two-tailed unpaired t-test**	**Summary**	**P value**
**control vs. Ift88^{DatCre}**	*	0.034
